# Prediction model using risk factors associated with anastomotic leakage after minimally invasive esophagectomy

**DOI:** 10.12669/pjms.39.5.8050

**Published:** 2023

**Authors:** Peng Su, Chao Huang, Huilai Lv, Zhen Zhang, Ziqiang Tian

**Affiliations:** 1 Peng Su, Department of Thoracic Fifth, Fourth Hospital of Hebei Medical University, Shijiazhuang 050011, Hebei Province, P.R. China; 2 Chao Huang, Department of Thoracic Fifth, Fourth Hospital of Hebei Medical University, Shijiazhuang 050011, Hebei Province, P.R. China; 3 Huilai Lv, Department of Thoracic Fifth, Fourth Hospital of Hebei Medical University, Shijiazhuang 050011, Hebei Province, P.R. China; 4 Zhen Zhang, Department of Thoracic Fifth, Fourth Hospital of Hebei Medical University, Shijiazhuang 050011, Hebei Province, P.R. China; 5 Ziqiang Tian, Department of Thoracic Fifth, Fourth Hospital of Hebei Medical University, Shijiazhuang 050011, Hebei Province, P.R. China

**Keywords:** Anastomotic leakage, Minimally invasive esophagectomy, Nomograph prediction model

## Abstract

**Objective:**

To explore the risk factors of anastomotic leakage after minimally invasive esophagectomy (MIE) and to build a prediction model of the probability of postoperative anastomotic leakage.

**Methods:**

Clinical data of patients undergoing MIE, admitted in the Fourth Hospital of Hebei Medical University from March 2018 to March 2022, were retrospectively selected, and risk factors of anastomotic leakage after MIE were analyzed by univariate and multivariate logistic regression. A prediction nomogram model was established based on the independent risk factors, and its prediction effect was evaluated.

**Results:**

A total of 308 patients were included. Thirty patients had postoperative anastomotic leakage, with an incidence of 9.74%. Logistic regression analysis showed that age, postoperative delirium, pleural adhesion, postoperative pulmonary complications, high postoperative white blood cell count and low lymphocyte count were risk factors for postoperative anastomotic leakage. A nomograph prediction model was constructed based on these risk factors. The predicted probability of occurrence of the nomograph model was consistent with the actual probability of occurrence. The calculated C-index value (Bootstrap method) was 0.9609, indicating that the nomograph prediction model had a good discrimination ability. By drawing the receiver operating characteristic (ROC) curve, we showed that the area under the curve (AUC) of the nomograph prediction model was 0.9609 (95%CI: 0.937-0.985), which indicated a good prediction efficiency of the model.

**Conclusions:**

The nomograph prediction model based on the independent risk factors of anastomotic leakage after MIE can accurately predict the probability of postoperative anastomotic leakage.

## INTRODUCTION

Esophageal cancer (EC) is one of the most common malignant tumor in the world, ranking fifth in China, and is associated with a high mortality rate and a poor prognosis.^1^-^3^ Surgery is considered a primary treatment for EC. Minimally invasive esophagectomy (MIE) is currently a procedure of choice in clinical practice,^4^,^5^ as it is generally safe and results in a radical resection.^6^ However, the incidence of postoperative complications after MIE can be high due to the anatomical characteristics of the esophagus.^5^,^6^ Among them, anastomotic leakage is the most common complication after MIE and is associated with prolonged hospital stay and increased risk of mortality.^7^ The treatment of anastomotic leakage is difficult, costly, is associated with prolonged hospitalization and may affect the long-term prognosis.^8^ In addition, patients after radical esophagectomy are at risk of rapidly developing thoracic infection, which may result in mediastinal infection. Subsequent complications, such as organ failure and septic shock, may lead to a 50%~70% mortality rate if not treated properly.^6^,^9^

Therefore, it is crucial to analyze the risk factors of postoperative anastomotic leakage to reduce the perioperative mortality of patients after MIE and improve their prognosis.^10^,^11^ At present, there are many reports about the factors related to anastomotic leakage after MIE, but there is no unified conclusion. As such, this study retrospectively selected patients who underwent MIE in our hospital. The main aim of this study was to further analyze the risk factors of anastomotic leakage after MIE and to build a prediction model of the probability of postoperative anastomotic leakage.

## METHODS

Clinical records of 308 patients (181 males and 127 females), who underwent MIE in the Fourth Hospital of Hebei Medical University from March 2018 to March 2022, were retrospectively collected. Anastomotic leakage occurred in 30 patients’ post-operation. Patients were divided into the Leakage group (n=30) and the non-leakage group (n=278) according to the presence of post-operative anastomotic leakage.

### Ethical Approval

This study was approved by the Ethics Committee of our hospital (No. 2022KY279, Date: 2022-05-07).

### Inclusion criteria:

- Patients diagnosed with EC.^12^
- Patients with primary tumor.
- Patients underwent MIE.^13^
- Patients with complete clinical data.

### Exclusion criteria:

- Those with a history of other malignant tumors.
- Those with other serious complications.
- Pregnant and lactating women.

### Observation indicators

Basic patient data, including gender, age, body mass index (BMI), hypertension, diabetes, arrhythmia, smoking history, postoperative delirium, pleural adhesion, postoperative pulmonary complications, pathological type, abdominal surgery history, and operation time.

Postoperative albumin index – albumin levels were measured by enzyme-linked immunosorbent assay (ELISA), using the kit from Shanghai Enzyme-linked Biological Co., Ltd. Postoperative white blood cell (WBC) and lymphocyte counts were measured by NH21 fully automated flow cytometry (Nuohai Life Science (Shanghai) Co., Ltd.)

### Statistical analysis

Data were analyzed using SPSS 26.0 and R 4.22. The counting data were expressed as frequency (percentage), and the chi-square test was used to compare the differences between groups. The Shaprio-wilk test and histogram were used to determine the normality of the measurement data, and non-normal distribution data were expressed as median and interquartile range. Mann-Whitney *U* test was used for inter group comparison. Statistical significance was considered at *P*<0.05. The receiver operating curve (ROC) was used to evaluate the diagnostic effectiveness of the prediction model. The Bootstrap self-sampling method (1000 repeated samples) was used to calculate accuracy and differentiation of the internal validation c-index test model. The calibration curve was used to evaluate the consistency between the predicted risk and the actual risk, and then the decision curve was used to analyze and evaluate the clinical efficacy of the risk model.

## RESULTS

There were no differences in gender, BMI, hypertension, diabetes, arrhythmia, smoking history, pathological type, BMI, and postoperative albumin between the two groups (*P*>0.05. Univariate analysis showed that age and WBC count post-operation in the Leakage group were higher, operation time was longer and the postoperative lymphocyte count was lower than the non-leakage group (*P*<0.05. The proportion of postoperative delirium, pleural adhesion and postoperative pulmonary complications in the Leakage group was higher than that in the non-leakage group (*P*<0.05. Table-I

**Table-I:** Single factor analysis of influencing factors of postoperative anastomotic leakage.

Variables	Leakage (n=30)	No-leakage (n=278)	χ^2^/Z	P
Male, n(%)	20(66.7)	161(57.9)	0.856	0.355
Age (year)	62.5(59, 68)	56(52, 62)	-5.387	<0.001
BMI (kg/m^2^)	22.5(21, 25)	23(21, 25)	-0.714	0.475
Hypertension, n(%)	10(33.3)	107(38.5)	0.306	0.580
Diabetes, n(%)	4(13.3)	31(11.2)	0.128	0.720
Arrhythmias, n(%)	2(6.7)	15(5.4)	0.084	0.772
Smoking history, n(%)	19(63.3)	170(61.2)	0.054	0.816
Postoperative delirium, n(%)	17(56.7)	85(30.6)	8.322	0.004
Pleural adhesion, n(%)	15(50.0)	65(23.4)	9.979	0.002
Postoperative pulmonary complications, n(%)	19(63.3)	90(32.4)	11.351	0.001
Pathological type - squamous cell carcinoma, n(%)	27(90.0)	260(93.5)	0.530	0.467
History of abdominal surgery	6(20.0)	33(11.9)	1.618	0.203
Operation time (hour)	4.25(3.9, 5.1)	3.7(3.2, 4.6)	-3.311	0.001
Postoperative albumin (g/L)	39(38, 41)	39(36, 41)	-1.239	0.215
Postoperative WBC count ( ×10^9^/L)	12.5(10.4, 13.5)	8.9(7.4, 10.5)	-6.617	<0.001
Postoperative lymphocyte count (×10^9^/L)	1.9(1.3, 2.1)	2(2, 2.5)	-4.007	<0.001

Multivariate logistic analysis showed that age, postoperative delirium, pleural adhesion, postoperative pulmonary complications, high WBC, and low lymphocyte count were independent risk factors for anastomotic leakage after MIE (OR=1.169; 4.143; 7.877; 5.650; 2.700; 0.095; *P*<0.05.Table-II

**Table-II:** Risk factors of anastomotic leakage after minimally invasive esophagectomy.

Variables	β	SE	Wald/χ^2^	P	OR	95% CI
Age	0.157	0.054	8.537	0.003	1.169	1.053~1.299
Postoperative delirium	1.422	0.642	4.904	0.027	4.143	1.178~14.579
Pleural adhesion	2.064	0.68	9.207	0.002	7.877	2.077~29.877
Postoperative pulmonary complications	1.732	0.652	7.062	0.008	5.650	1.575~20.262
Postoperative WBC count	0.993	0.223	19.787	<0.001	2.700	1.743~4.183
Postoperative lymphocyte count	-2.352	0.837	7.903	0.005	0.095	0.018~0.491

**Fig.1:**
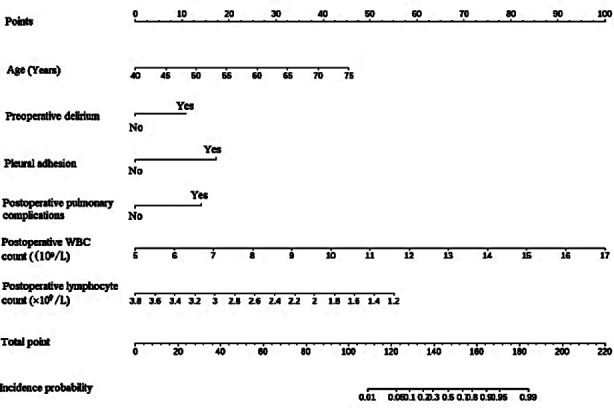
Risk model of anastomotic leakage after minimally invasive esophagectomy.

**Fig.2:**
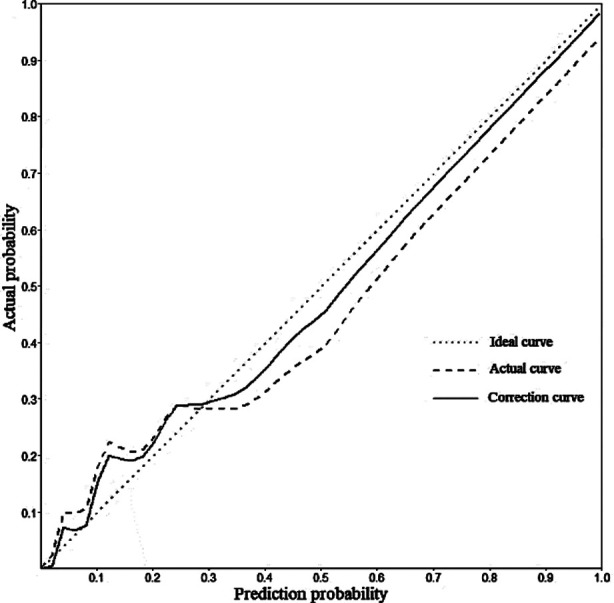
Calibration curve of nomograph model.

**Fig.3:**
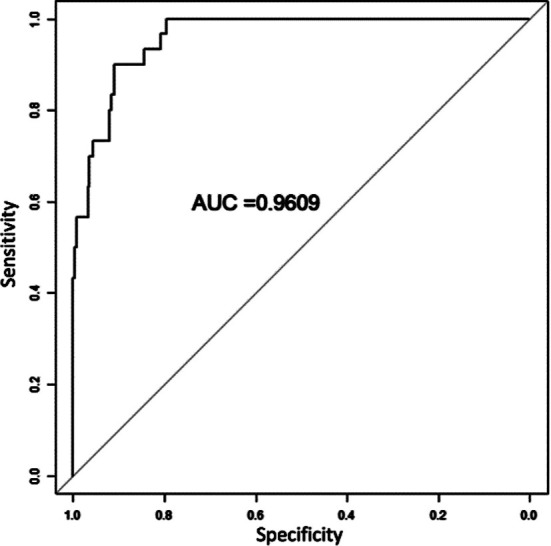
ROC curve of nomograph prediction model.

**Fig.4:**
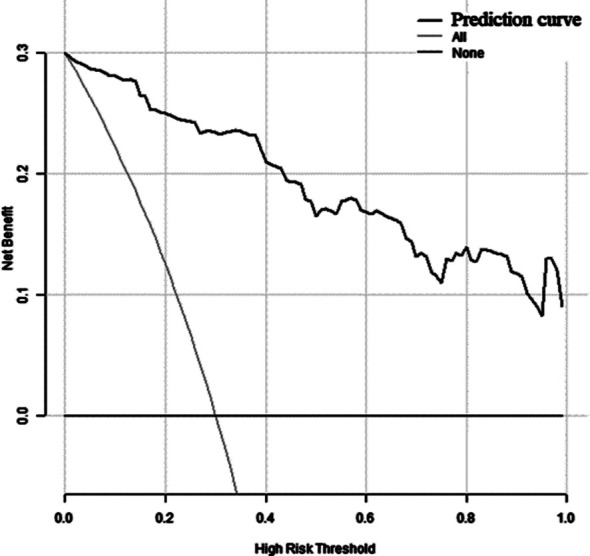
Decision curve analysis of nomograph prediction model

Based on the independent risk factors screened out by the multivariate logistic regression analysis, R software 4.22 and its rms package were used to build a nomogram model for predicting the risk of anastomotic leakage after MIE (Fig.1). The goodness of fit of the prediction model was evaluated by the Hosmer-Lemeshow method (*χ^2^*=2.178, *P*=0.975). The Bootstrap self-sampling method was then used to calculate the differentiation of the prediction model to carry out internal verification. Bootstrap repeated sampling (1000 times) to obtain the calibration curve of the model, and demonstrated that the predicted coincidence probability of the model was consistent with the actual probability of occurrence (Fig.2). The calculated C-index value was 0.9609, which showed a good distinguishing ability of the model. By drawing the ROC curve, we showed that the AUC of the ROC curve of the nomograph prediction model was 0.9609 (95% CI: 0.937-0.985) (Fig.3), which proved that the model had a good prediction efficiency and a good discrimination ability. The analysis of the drawing of the decision curve indicated the high value of this prediction model (Fig.4).

## DISCUSSION

Our results showed a lower probability of anastomotic leakage after MIE (9.74%; 30/308), which is consistent with previous reports. A study if Huang et al. showed that in 544 patients with esophageal and cardiac cancer who underwent surgery, an incidence of anastomotic leakage was 9.2% (50/544).^14^ Similarly, Li et al. demonstrated that in 1257 EC patients who underwent esophagectomy and intrathoracic anastomosis, anastomotic leakage incidence was 7.8%.^15^ However, other reports showed significantly lower (11.4% - 21.2%) probability of anastomotic leakage after radical resection of EC.^16^ One study, including 174 patients with EC, found that the incidence of anastomotic leakage after the operation was 18.96%.^17^ These conflicting results may be caused by the geographical differences.

Our results also showed that age, postoperative delirium, pleural adhesions, postoperative lung complications, higher WBC count and low lymphocyte count were risk factors for anastomotic leakage after MIE. Among them, factors such as older age are consistent with the current literature, suggesting that older EC patients have a progressive decline in general physiological function. As a results, this population may be negatively affected by the stress, and is more likely to develop postoperative anastomotic leakage.^15^ Liu YT et al^18^ suggested that the risk factors of postoperative anastomotic leakage in cancer patients include gender, high body mass index, malnutrition, smoking, tumor diameter >2.5cm, intraoperative hypothermia, high level of tumor markers, and long operation time. However, our results showed that the operation time was not a risk factor for postoperative anastomotic leakage, which may be related to the selection bias of sample size.

Previous studies identified gender, age, body mass index, postoperative complications, etc as the risk factors of anastomotic leakage after MIE.^15^,^17^,^18^ However, other factors, included in the current study, such as postoperative delirium, adhesion of the thoracic cavity at the operative side, WBC count of the whole blood after the operation and lymphocyte count after the operation, are rarely considered.^19^,^20^ Some patients with postoperative delirium do not cooperate with postoperative recovery, and can be prone to hypoxemia. This could impair the diffusion function of the patient’s lungs, resulting in organ and tissue hypoxia or even respiratory failure. This, in turn, may lead to lung inflammation, frequent cough and expectoration, which can increase anastomotic stoma tension, resulting in an increase in the incidence of postoperative anastomotic leakage.^21^,^22^

Adhesion of the thoracic cavity on the surgical side not only increases the difficulty of thoracic surgery, but also prolongs the operation time, and to some extent affects tissue healing and the long-term survival rate.^23^,^24^ Patients with postoperative pulmonary complications have a frequent and aggravated cough due to pulmonary infection, which increases the tension at the anastomotic site, thus increasing the risk of anastomotic leakage.^22^ Lymphocyte count is an important indicator of the nutritional status of the body. Low lymphocyte count can lead to decreased immunity, disruption of the autoimmune system and increased possibility of postoperative anastomotic leakage.^23^ Based on our results, special attention should be paid to the EC patients who are older, experienced delirium after the operation, have adhesion of thoracic cavity at the operation side, suffer from postoperative pulmonary complications, and have high WBC and low lymphocyte count after the operation. Active treatment measures should be taken to reduce the probability of anastomotic leakage after MIE.^22^-^24^

This study used univariate analysis and multivariate logistic regression analysis, to establish a nomograph prediction model prediction model of the risk of anastomotic leakage after MIE. Analysis of the Hosmer-Lemeshow method, calibration curve, C-index value, ROC curve, AUC=0.9609 and decision curve demonstrated that the proposed prediction model is accurate and valuable. This model allows personalized evaluation of patients undergoing MIE, making it more convenient and intuitive for clinicians. This model may be used to quantify the risk of postoperative anastomotic leakage in EC patients, and provide a reference basis for the formulation of perioperative treatment plan.

### Limitation of the study

This is a single-center retrospective cohort study. Therefore, only single-center data was used in model validation, and multi-center model validation was not conducted. Further studies with more factors and laboratory indicators are needed. Additionally, prospective cohort studies should be carried out to further adjust and optimize the values of the model and to make it more accurate and more in line with clinical practice.

## CONCLUSION

Our study showed that age, postoperative delirium, adhesion of the thoracic cavity at the operative side, postoperative pulmonary complications, high WBC after operation and low lymphocyte count after operation are independent risk factors of anastomotic leakage after MIE. Such nomogram prediction model based on the independent risk factors can accurately evaluate the probability of postoperative anastomotic leakage.

### Authors’ Contributions:

**PS:** Conceived and designed the study.

**CH**, **HL**, **ZZ** and **ZT:** Collected the data and performed the analysis.

**PS:** Was involved in the writing of the manuscript and is responsible for the integrity of the study.

All authors have read and approved the final manuscript.
